# New Pathophysiological Aspects of Growth and Prevention of Kidney Stones

**DOI:** 10.1155/2012/175843

**Published:** 2012-05-20

**Authors:** J. M. Baumann, B. Affolter

**Affiliations:** Laboratories Viollier, Departement of Stone Research, Gartenstrasse 9, 2502 Biel, Switzerland

## Abstract

Kidney stones probably grow during crystalluria by crystal sedimentation and aggregation (AGN) on stone surfaces. This process has to occur within urinary transit time (UT) through the kidney before crystals are washed out by diuresis. To get more information, we studied by spectrophotometry the formation and AGN of Ca oxalate (Ca Ox) crystals which were directly produced in urine of 30 stone patients and 30 controls by an oxalate (Ox) titration. Some tests were also performed after removing urinary macromolecules (UMs) by ultrafiltration. To induce rapid crystallization, high Ox additions (0.5–0.8 mM) were necessary. The most important finding was retardation of crystal AGN by UM. In urine of 63% of controls but only 33% of patients, no AGN was observed during an observation of 60 minutes (*P* < 0.05). Also growth and sedimentation rate of crystals were significantly reduced by UM. For stone metaphylaxis, especially for posttreatment residuals, avoiding dietary Ox excesses to prevent crystal formation in the kidney and increasing diuresis to wash out crystals before they aggregate are recommended.

## 1. Introduction

After minimal invasive treatment, stone residuals are found in 20–25% of patients [1, 2]. These stone fragments are an interesting model to study stone growth *in vivo*. Depending on observation time, 20–37% of stone residuals show further growth which can lead to complications and secondary interventions [2, 3]. On the other hand, in a retrospective study, the size of isolated and not infected stone residuals after extracorporeal shock wave lithotripsy (ESWL) remained stable in 24 of 83 patients even during an average observation time of 3.5 years [3]. These observations suggest that stone formation occurs only periodically. Therefore, questions arise about factors inducing stone growth and about rational measures of prevention.

Scanning microscopy of kidney stones shows within an organic matrix a highly aggregated crystal structure [4]. Already in 1969, it was demonstrated that stone patients have a tendency to excrete large crystal aggregates [5]. In the meantime, many indices were found that crystal aggregation (AGN) is an important process in stone formation [6] and especially in stone growth [7]. In urine, crystals are always coated by a layer of urinary proteins [8], which essentially influence crystallization processes. Main urinary proteins thought to be involved in stone formation are albumin, inter-alpha-inhibitor, nephrocalcin, osteopontin, prothrombin fragment 1, and Tamm Horsfall protein or uromodulin. Glycosaminoglycans seem to be of minor importance [9]. Much work was done to evaluate the influence of these substances on crystallization processes especially of Ca oxalate (Ca Ox), the predominant stone mineral [9, 10]. However, results were controversial, and the impact on stone prevention remained minimal [10]. The three crystallization processes, nucleation, crystal growth, and AGN, often were separately examined in different test systems [11, 12]. Tests were often performed in artificial solutions with the addition of urine in high dilution or of previously isolated urinary proteins [6, 13]. AGN generally was studied using preformed crystals. These crystals were, for reasons of reproducibility, produced in protein-free solutions and ripened for several hours or days [14, 15]. However, AGN being relevant for stone formation bases on freshly precipitated crystals which are coated by urinary proteins and which aggregate within the short transit time of crystals through the kidney and the urinary tract [16, 17]. Therefore, we tried to study the formation and AGN of Ca Ox crystals which we directly produced in urine by an Ox titration. Crystallization was continuously observed by spectrophotometry, and some sediments were analyzed by scanning electron microscopy [17, 18]. Recently, these crystallization tests were performed in urine of stone patients and controls to get more information about general aspects of crystal AGN [19]. In this paper, results of the mentioned study were evaluated especially with respect to stone growth and were completed by repeating some tests after removing urinary macromolecules by ultrafiltration.

## 2. Material and Methods

### 2.1. Collection of Urine and Preparation of Urine and Control Solution

Urine was collected from 30 patients (24 men and 6 women aged 16–79, mean 52 years) prior to undergoing ESWL for a calcium stone and therefore being under similar conditions like patients with stone residuals after minimal invasive treatment. Urine of controls was collected from 30 patients without a history of urological or nephrological disease (22 men and 8 women aged 16–80, mean 52 years) prior to undergoing hand surgery. Patients and controls being on free diet were fasting at least two hours before starting the urinary collection period, which exactly was recorded. Urine volume was determined, and urine with a pathology on stick examination (Combur Test UX, Roche, Switzerland) was excluded with the exception of microhematuria which is frequent in patients with a stone *in situ* and therefore also representative for the situation of stone growth.

To obtain comparable results, our intention was to perform crystallization tests at almost identical states of supersaturation. Therefore, from the collection period, a volume corresponding to a diuresis of 100 mL/h was calculated, and all urines were diluted with distilled water to a volume corresponding to this diuresis. This dilution allowed to equalize differences of urine composition due to different states of diuresis and to adapt all samples to the same ionic Ca and sodium concentration before performing crystallization tests. Also endogenous urinary oxalate concentration was minimized being after dilution 0.13 ± 0.05 mM in patients and 0.08 ± 0.03 mM in controls. A control solution was prepared by buffering distilled water with 5 mM sodium cacodylate.

### 2.2. Crystallization Study

pH was adjusted in all samples to 6.0. In urine, Ca and sodium concentrations were determined by an ion-selective electrode (AVL List GmbH, Graz, Austria). In urine and in control solution (CS), ionic Ca was adjusted to 2.0 mM and sodium to 100 mM. 2 mL of urine, and CS were placed in a quartz micro cuvette within a thermostat-table cell holder of a Perkin Elmer spectrophotometer 550S (Perkin Elmer, Rotkreuz, Switzerland) at 37°C. Under continuous stirring, Ox concentration was raised by 0.1 mM sodium oxalate per minute up to a final addition of 1.5 mM. High Ox concentrations were used because in a previous study after addition of 1.0 mM Ox to urine of healthy controls no AGN was found [17]. Urinary Ox was not taken into account since it was even in 90% of stone patients less than 10% of the Ox addition.

During and after oxalate titration, optical density (OD)—reflecting the concentration of particles being suspended in urine and control solution—was monitored by the spectrophotometer at 620 nm wavelength. From the time where an increase of OD was observed and from titration rate, the critical Ox addition necessary to induce crystal formation, called metastable limit, was calculated. At the end of Ox titration, maximal OD (mOD) was measured, and stirring was stopped. The decrease of OD (dOD/dt, min^−1^) reflecting crystal sedimentation and AGN was followed during a further observation time of 60 minutes. From mOD and initial dOD/dt, a sedimentation rate (SR, mm/min) was calculated by the following equation where *L* represents the height of the light beam (7 mm) in the spectrophotometer:

(1) $$\text{SR}=\frac{L\times \left(\text{dOD}/\text{dt}\right)}{\text{mOD}}$$

crystallization tests were repeated after ultrafiltration of urine of 10 healthy controls in a hemodialyser (Hemoflow F3, Fresenius AG, Bad Homburg, Germany), the exclusion limit of the dialysis membrane being 5 kD.

### 2.3. Statistics

Probabilities were calculated by Mann-Whitney *U* test and by Fisher's test.

## 3. Results

**Figure 1:**
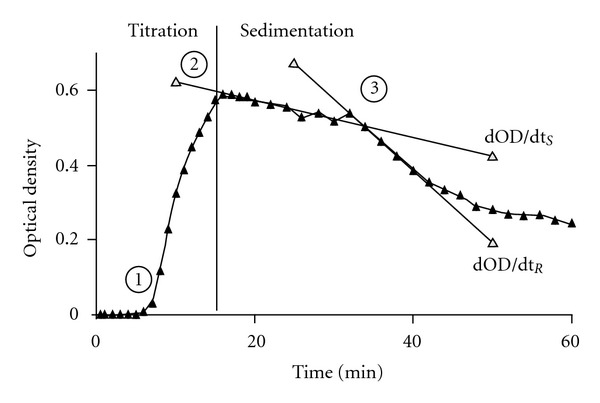
Spectrophotometric crystallization curve:  (1) start of increase of optical density (OD) reflecting crystal concentration in urine, (2) maximal OD (mOD) reached at the end of oxalate titration, (2)-(3) slow OD decrease (dOD/dt_*S*_) by sedimentation of single crystals, and (3) start of rapid OD decrease (dOD/dt_*R*_) indicating crystal aggregation.

Figure 1 shows a typical spectrophotometric crystallization curve which can be separated into a titration and a sedimentation phase. After 6 min of Ox titration corresponding to an addition of 0.6 mM sodium oxalate, optical density (OD) started to increase (Pt. 1). From this critical Ox addition called metastable limit (ML) and the initial urinary Ox concentration in the test system, the critical supersaturation being necessary to induce crystal nucleation can be calculated. Pt. 2 marks the maximal OD or crystal concentration, respectively, reached by the Ox titration. After the end of the titration where stirring was stopped, a phase of slow OD decrease started almost immediately (Figure 1, Pt. 2-3). In previous studies, this slow OD decrease could be attributed by scanning electron microscopy (SEM) of the corresponding sediments to the sedimentation of single crystals of Ca Ox monohydrate [17, 18]. After a certain time (Pt. 3) called suspension stability (SS), a rapid decrease of OD was observed, which indicates AGN since sediments obtained after such rapid OD decreases showed large crystal aggregates in high concentration [17, 18].

**Figure 2:**
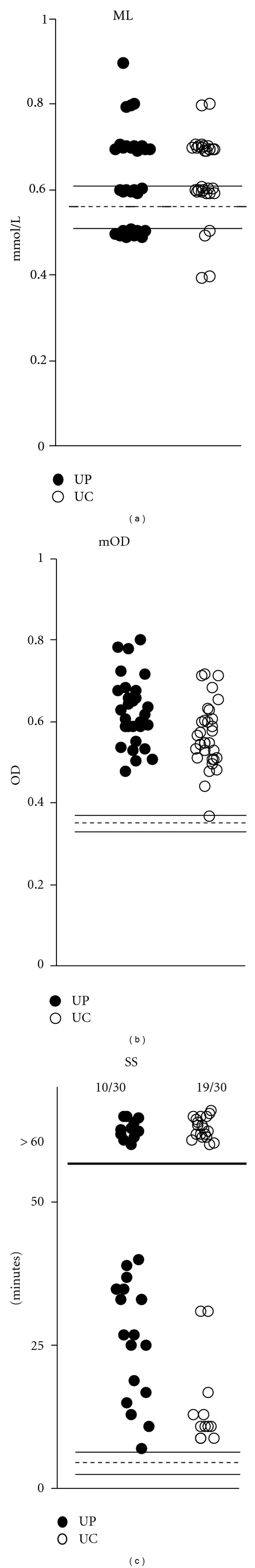
Crystallization parameters in urine of stone patients (UPs) and of controls (UCs) and in control solution (shaded area, mean ± SD): ML (metastable limit = Ox addition necessary to induce crystallization), mOD (maximal optical density reached at the end of Ox titration), and SS (suspension stability = time after titration without aggregation).

In Figure 2, metastable limit (ML), maximal OD (mOD), and suspension stability (SS) observed in urine of stone patients (UP), in urine of healthy controls (UC), and in control solutions (CS) were compared. Mean values of ML were significantly higher in UP and UC than in CS (*P* < 0.01) demonstrating that in UP and UC due to inhibition of crystal nucleation despite the presence of urinary Ox, more Ox addition was necessary to induce crystallization. Also mOD reflecting crystal concentration after titration was in UP and UC significantly higher than in CS (*P* < 0.001). Since initial supersaturation and crystal deposit (on average 1.35 mM) were almost identical in all experiments, an increased mOD means that in UP and UC under identical conditions more and smaller crystals were produced than in CS. This effect can only be explained by an inhibition of crystal growth. However, the most interesting finding was made with respect to suspension stability (SS). A group of UP as well as of UC showed compared to CS a significantly higher SS or retardation of AGN, respectively (*P* < 0.001). In a second group of UP and UC even after an observation time of 60 min, no signs of AGN were found. Interestingly, this absence of AGN was more frequent in urine of healthy controls than of stone patients (63% versus 33%, *P* < 0.05).

**Figure 3:**
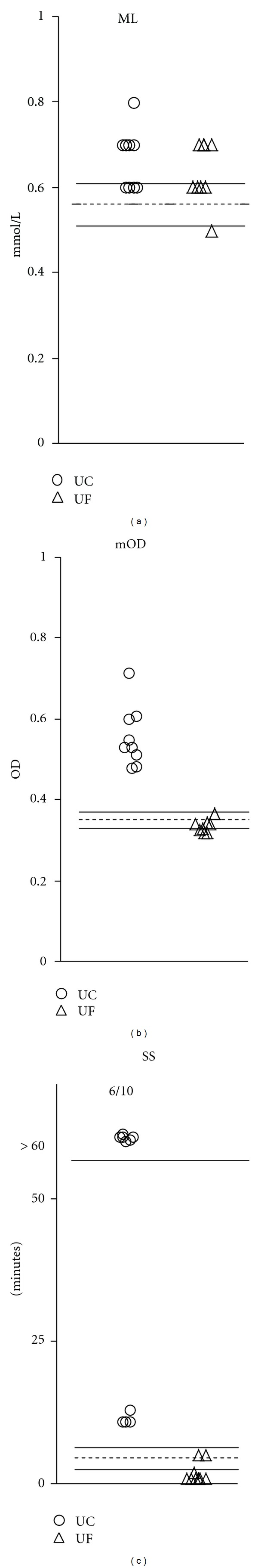
Crystallization parameters (see Figure 2) in urine of controls (UCs), in ultrafiltrate of the same urine (UF), and in control solution (shaded area).

To get more information about the source of urinary inhibition of nucleation, growth, and AGN, we compared metastable limit (ML), maximal OD (mOD), and suspension stability (SS) observed in urine of 10 controls before and after ultrafiltration of urine by a membrane with an exclusion limit of 5 kD. Results of urine of controls (UCs) containing urinary macromolecules >5 kD (UM) and ultrafiltrate (UF) containing only low-molecular-weight compounds were compared with results obtained in the control solution (CS).  Figure 3 shows that inhibition of crystal nucleation can be attributed to low-molecular-weight compounds of urine since no significant difference was found with respect to ML between UC and UF. On the other hand, elevated values of mOD indicating inhibition of crystal growth and of SS representing retardation of AGN were exclusively found in UC and therefore seems to be caused by UM. 

**Figure 4:**
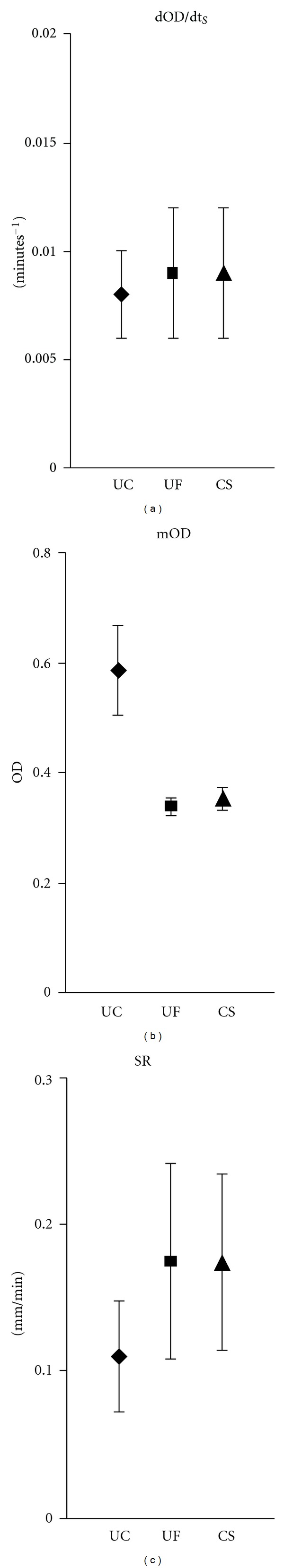
Slow OD decrease (dOD/dt_*S*_), maximal optical density (mOD), and sedimentation rate of single crystals (SRs) in urine of controls (UCs), in ultrafiltrate of the same urine (UF), and in control solution (CS), mean ± SD.

For stone growth, crystals have to accumulate on stone surfaces which *in vivo* mainly seems to occur by sedimentation [18]. Therefore, we calculated the sedimentation rate of single crystals (SR) in UC, UF, and CS from slow dOD/dt and mOD using (1). In Figure 4, mean values ± SD of dOD/dt_*S*_, mOD, and SR are listed. UC showed compared to UF and CS a significantly lower SR (*P* < 0.01). Since dOD/dt_*S*_ values were nearly identical in the three groups, the low SR of UC could exclusively be attributed to the significantly elevated mOD (*P* < 0.001) which, as explicated above, indicates a decreased crystal size. This finding is in agreement with the rule of stoke that sedimentation rate increases as a function of particle diameter in square and confirms that UM mainly seems to be responsible for the inhibition of crystal growth.

## 4. Discussion

Retardation of crystal aggregation (AGN) by urinary macromolecules (UMs) was the most important finding when studying Ca Ox crystallization directly in urine. Although crystallization was induced by high Ox concentrations, urine of 63% controls but only of 33% stone patients showed no signs of AGN during an observation time of 60 minutes. In the remaining urines, AGN occurred with an average delay of 20 minutes. For stone growth, AGN between crystals being suspended in urine and kidney stones has to occur within urinary transit time (UT) through the renal pelvis, before crystals are washed out by diuresis. Therefore, retardation of AGN seems to be a natural mechanism to prevent stone growth. Average UT in renal pelvis being reciprocal to diuresis was calculated dividing average volume of renal pelvis of 7 mL by an average diuresis of 0.6 mL/min and found to be in the range of 12 minutes [16]. Retardation of AGN by UM together with other factors discussed below may explain why stone formation is limited to about 10% of the population and has a recurrence rate of only 0.10–0.15 stones/patient and year [20], although urinary supersaturation especially with respect to Ca oxalate and crystalluria is common.

Rational stone metaphylaxis should either intend to prolong the retardation of AGN or to reduce UT in order that crystals are washed out before they can aggregate with stones or stone fragments. Retardation of AGN seems to base on urinary macromolecules (UMs) which coat urinary crystals [8]. These UM coats separate crystals in a distance where Van der Waal's forces, generally thought to be responsible for AGN, become extremely week [21]. Other mechanisms of AGN are therefore discussed. AGN in urine could base on viscous binding by pathological UM [13] or on Ca bridges between electronegative UM coats of the crystals [22]. UM cannot therapeutically, with the exception of rare cases of pathological Tamm Horsfall protein [23], be influenced until now. Experiments to prolong the delay of AGN by the addition of high doses of citrate or pyrophosphate to UM have failed [19]. However, UT in the renal pelvis, as the calculation above shows, can efficiently be reduced by an increase of diuresis or of fluid intake, respectively. The fear that increased diuresis by dilution of crystallization inhibitors has a negative effect on crystallization conditions seems not to be justified [24]. Also in our study, a high inhibition of crystal growth and AGN was found despite of urine dilution corresponding to a diuresis of 100 mL/hour.

Crystal accumulation on stone surfaces is another important factor for stone growth during crystalluria. Studies of crystal diffusion and sedimentation revealed that *in vivo* sedimentation seems to be the most efficient mechanism for crystal accumulation on stone surfaces [18]. Crystal accumulation on stone surfaces (mmol/cm^2^ and min) can be calculated multiplying crystal concentration (mmol/cm^3^) by sedimentation rate of crystals (cm/min). In urine, due to the inhibition of crystal growth by UM, relative low sedimentation rates were found, which like SS therapeutically can not be influenced until now. Reducing urinary supersaturation with respect to stone minerals together with an increase of diuresis which also reduces supersaturation remains therefore the base of every rational stone metaphylaxis. These measures can also prevent crystal formation in the kidney which occurs in distal segments of the nephron where UT is in the order of 1–1.5 minutes [25, 26]. In our experiments, Ox additions of 0.5–0.8 mM were necessary to induce rapid crystal nucleation. In nonhyperoxaluric patients, such high Ox concentrations are only observed in urine after excessive ingestion of Ox-rich food [27]. Dietary excesses with chocolate, ice tea, and other products with a high Ox content should therefore strictly be avoided. In a recent study, medical treatment by alkaline citrate was found to significantly reduce the formation of new stones and the growth of stone residuals after ESWL or percutaneous nephrolithotomy. However, the incidence of patients with hypocitraturia was with 43% extremely high in this study [28]. Citrate is an excellent chelator of Ca which can reduce ionic Ca concentration below the value of 1.2 mM found to be essential for AGN [29] but otherwise at least in our experiments had no direct influence on crystallization conditions [19]. In another study performed in 100 patients with stone residuals after ESWL, thiazide reduced the incidence of a secondary intervention from 42% to 18% [30]. However, it is not clear whether this beneficial effect can be attributed to a stabilization of stone growth by reducing urinary Ca concentration or mainly to an accelerated elimination of stone fragments due to an increase of diuresis.

## 5. Conclusions

There is evidence that kidney stones grow by crystal AGN on stone surfaces during crystalluria. To aggregate, crystals have to settle to the stone surfaces by sedimentation. Sedimentation rate increases with increasing particle size. Therefore, urinary macromolecules (UMs) inhibiting crystal growth and decreasing sedimentation rates are an important factor preventing stone growth. Another even more important factor is the ability of UM to retard AGN until crystals, being suspended in urine, are washed out of the kidney by diuresis. Rational prevention of stone growth therefore tends to increase diuresis and to avoid excessive oxalate intake which can provoke crystal formation already in the kidney.
